# Supplementary material for: Treatment of Metastatic Melanoma at First Diagnosis: Review of the Literature
Source: Life (Basel). 2022 Aug 24;12(9):1302. doi: 10.3390/life12091302 (PMC9505710; doi:10.3390/life12091302)
Supplement: Supplementary file 1 [file life-12-01302-s001.zip › life-1809998-supplementary.pdf]

Supplementary:

Initially we tried to do a systematic review of the literature, but the lack of results led to the article being presented without the systematic review.

Methods:

A systematic review was conducted in accordance with the PRISMA 2020 Declaration Guidelines. A literature search was performed through PubMed and electronic databases. The text words "first diagnosis" "first presentation" with the use of the Boolean operator "AND" "Metastatic Melanoma", "Stage IV Melanoma" was used to identify studies that discussed the efficacy of the drug in patients with metastatic melanoma.

The search strategy is shown in the PRISMA flowchart (Figure S1).
The following inclusion criteria were used:
(1) article within the last 20 years
(2) must be an academic or peer-reviewed source, and
(3) articles available in the English language.
Exclusion criteria were the following:
(1) articles prior to 2002, and
(2) articles not available in English.
Data extraction and analysis was performed by the authors and 14 studies were selected.

The methodological quality of each included study was assessed based on the Cochrane risk of bias tool.

The Cochrane GRADE (Grading of Recommendation Assessment, Development and Evaluation) approach assessed eight factors for the certainty of evidence for each outcome: (a) Risk of bias, (b) Inconsistency, (c) Imprecision, (d) Directionality, (e) publication bias, (f) dose-response relationship, (g) effect size, and (h) confounding. Articles were graded as follows: (1) well conducted randomized controlled clinical trial (RCCT); (2) well-designed controlled trial without randomization or prospective comparative cohort studies; (3) case-control studies, or retrospective cohort studies; (4) cross-sectional study; and (5) case series or observational studies (clinical or experimental).

Results:

The main studies analyzed are included in tables 2 and 3 (Manuscript). The steps for their selection are shown in Figure S1.

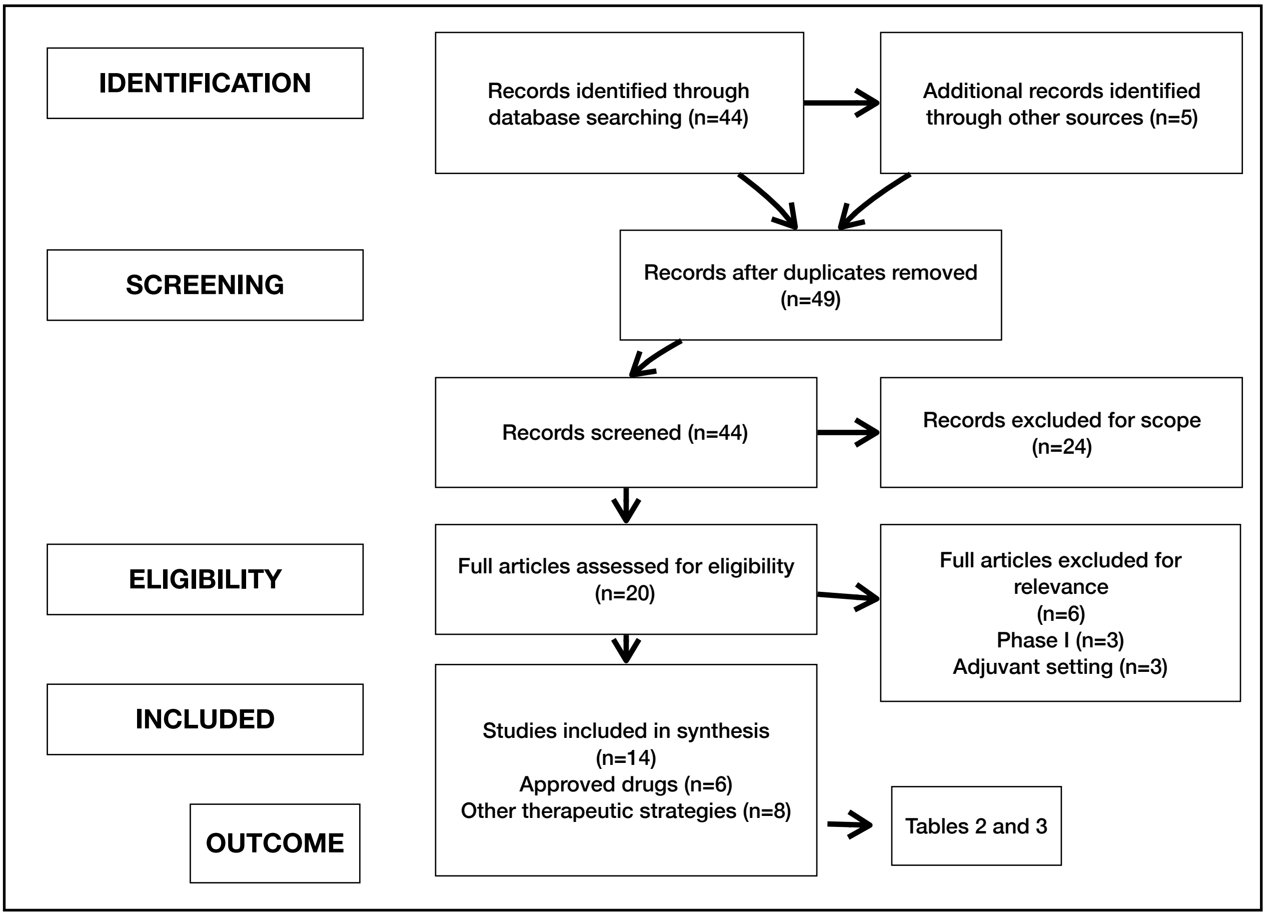

Figure S1. PRISMA flow chart for search strategy
